# Defining and Implementing Value-Based Healthcare for Older People from a Geriatric and Gerontological Perspective

**DOI:** 10.3390/ijerph191811458

**Published:** 2022-09-12

**Authors:** Yoshihisa Hirakawa

**Affiliations:** Department of Public Health and Health Systems, Nagoya University Graduate School of Medicine, Nagoya 466-8550, Japan; y.hirakawa@med.nagoya-u.ac.jp; Tel.: +81-52-744-2128

The world’s population is ageing at a faster rate than ever before; it is estimated that there are currently over 1 billion people aged 60 years or older, mostly living in low- and middle-income countries [1]. Developing strategies for improving the quality of life and promoting the well-being of individuals as they age within their family, community, and society is vital to lessen the impact of global population ageing. Thus, global action on healthy ageing is urgently needed to ensure that older people can fulfill their potential with dignity and equality in a healthy environment. Researchers and practitioners in this field aim to improve health care value and associated meaningful person-centered outcomes through interdisciplinary collaboration in geriatrics and gerontology, including diagnosis, prevention, treatment, management of multi-morbidity, and end-of-life care [2]. Therefore, researchers and practitioners in geriatrics and gerontology should serve as a bridge between health care system operations and health services research to promote a culture of value-based, data-driven decision making. To achieve that, facilitating collaborative interaction among multidisciplinary stakeholders to generate and implement innovative, scientifically grounded and technology-based solutions is required in geriatrics and gerontology [3]. The effective translation of health care research findings into policy and geriatric care practices could lead to high-value geriatric care worldwide.

For older people aged 65 to 75, good health helps ensure independence, security, and continued productivity in their later years. Non-communicable diseases (NCDs) such as cancer, cardiovascular disease, diabetes, and COPD have now become the largest cause of mortality and disability globally for this age group [4]. NCDs are known to diminish older people’s quality of life, raise health care costs, and add pressure for family members who are responsible for their care. Primary health care is essential for the prevention and treatment of NCDs through the management of risk factors and coordination of care and medications [5]. A large body of research has suggested that the management and prevention strategies for NCDs and their risk factors are fundamentally different for older and younger people. The Japanese Geriatrics Society, in conjunction with other relevant professional societies, has published clinical guidelines for NCDs such as diabetes and hypertension [6] that consider the differences between older and younger patients. However, there are important challenges that need to be overcome to ensure the successful implementation of these guidelines for older patients, including decreased adherence to physician’s advice and instructions due to physical and cognitive impairment, as well as limited access to formal and informal support, for financial and social reasons such as living alone or in poverty, and to health information, partly due to the digital divide.

Because care for older people is often provided by a number of different professionals, a collaborative and interprofessional framework for the prevention and management of frailty is gaining increasing interest among health and social care professionals, scientists, public health experts and care planners [7]. Interprofessional education (IPE) helps develop and promote interprofessional thinking and acting. IPE is a model in which individuals from two or more health care professions learn together during all or part of their professional and postgraduate training with the goal of cultivating interprofessional practice [8].

However, a number of studies have indicated that the implementation of interprofessional practices still poses various challenges [9]. Interprofessional practices can be compromised when healthcare professionals are not aware of their benefits for older clients, or when they are too busy to implement them. Hierarchy among health professionals is also one of the most widely recognized barriers to the implementation of interprofessional practices [10]. Hierarchy can be a source of conflict or poor communication among healthcare professionals partly because of the disparity in values that separate them. The ethics and values of interprofessional collaboration (IPC) and hierarchy-related barriers need to be rationally explained, and thus, it is crucial to gain a comprehensive view about what hinders and facilitates the practice of IPC.

Clinical practice guidelines have become widely used to guide quality improvement of clinical practice; these are systematically developed statements comprising frameworks for clinical decisions and supporting best practices, which include recommendations intended to optimize patient care. Guidelines designed for older people aim to support a person-centered approach to improve quality of life. Compared to younger people, older people are likely to have more chronic medical conditions and to exhibit widely heterogeneous health status, ranging from robust to very severely frail. This heterogeneity and individual medical complexity make standardized care for older patients particularly challenging, requiring a deep understanding of the patient’s personal values and goals. Most current healthcare guidelines are disease-specific and do not adequately address this complexity and heterogeneity, thus limiting their implementation for older patients.

As mentioned above, a number of guidelines for improving the care of older people have been developed around the world to specifically address this complexity and provide guidance to physicians and help them prioritize disease-specific therapies and goals for their patients. Providing a rationale for prioritizing recommendations and the inclusion of multifactorial conditions prevalent in older people could contribute to the development of a model for clinical guidelines involving a multidisciplinary team. Qualitative research may be a useful way to improve the quality and implementation of such guidelines [11,12]. The qualitative evidence methodology used in guideline development is a systematic review of multiple primary qualitative studies that bring together findings from different studies to offer new and broad understandings of social and psychological barriers and facilitators and draw comprehensive recommendations for clinical guidelines involving a multidisciplinary team.

Researchers and practitioners in geriatrics and gerontology may serve as a bridge between health care system operations and health services research to promote a value-based, data-driven decision-making culture. This can be achieved by establishing a respectful, low-conflict environment among professionals that fosters good communication and tolerance for disparities in values and health status among older patients. To conclude, the results of qualitative systematic reviews of multiple primary qualitative studies can contribute to the provision of a new, broader understanding of social and psychological barriers and facilitators to comprehensive recommendations for clinical guidelines involving a multidisciplinary team.
